# Diagnostic utility of 7T neuromelanin imaging of the substantia nigra in Parkinson’s disease

**DOI:** 10.1038/s41531-024-00631-3

**Published:** 2024-01-08

**Authors:** Dhairya A. Lakhani, Xiangzhi Zhou, Shengzhen Tao, Vishal Patel, Sijin Wen, Lela Okromelidze, Elena Greco, Chen Lin, Erin M. Westerhold, Sina Straub, Zbigniew K. Wszolek, Philip W. Tipton, Ryan J. Uitti, Sanjeet S. Grewal, Erik H. Middlebrooks

**Affiliations:** 1 Department of Radiology, Johns Hopkins University, Baltimore, MD USA; 2 Department of Radiology, Mayo Clinic, Jacksonville, FL USA; 3 Department of Biostatistics, West Virginia University, Morgantown, WV USA; 4 Department of Neurology, Mayo Clinic, Jacksonville, FL USA; 5 Department of Neurosurgery, Mayo Clinic, Jacksonville, FL USA

**Keywords:** Diagnostic markers, Parkinson's disease

## Abstract

Parkinson’s disease (PD) is a prevalent neurodegenerative disorder that presents a diagnostic challenge due to symptom overlap with other disorders. Neuromelanin (NM) imaging is a promising biomarker for PD, but adoption has been limited, in part due to subpar performance at standard MRI field strengths. We aimed to evaluate the diagnostic utility of ultra-high field 7T NM-sensitive imaging in the diagnosis of PD versus controls and essential tremor (ET), as well as NM differences among PD subtypes. A retrospective case-control study was conducted including PD patients, ET patients, and controls. 7T NM-sensitive 3D-GRE was acquired, and substantia nigra pars compacta (SNpc) volumes, contrast ratios, and asymmetry indices were calculated. Statistical analyses, including general linear models and ROC curves, were employed. Twenty-one PD patients, 13 ET patients, and 18 controls were assessed. PD patients exhibited significantly lower SNpc volumes compared to non-PD subjects. SNpc total volume showed 100% sensitivity and 96.8% specificity (AUC = 0.998) for differentiating PD from non-PD and 100% sensitivity and 95.2% specificity (AUC = 0.996) in differentiating PD from ET. Contrast ratio was not significantly different between PD and non-PD groups (*p* = 0.07). There was also significantly higher asymmetry index in SNpc volume in PD compared to non-PD cohorts (*p* < 0.001). NM signal loss in PD predominantly involved the inferior, posterior, and lateral aspects of SNpc. Akinetic-rigid subtype showed more significant NM signal loss compared to tremor dominant subtype (*p* < 0.001). 7T NM imaging demonstrates potential as a diagnostic tool for PD, including potential distinction between subtypes, allowing improved understanding of disease progression and subtype-related characteristics.

## Introduction

Parkinson’s disease (PD) is the second most common neurodegenerative disorder and may present with a variety of motor and non-motor symptoms^1^. A pathologic hallmark of PD is degeneration of dopaminergic cells in the substantia nigra pars compacta (SNpc). The SNpc normally accumulates neuromelanin (NM), which is an oxidative byproduct of the metabolism of dopamine and norepinephrine^2–4^. The NM pigment is only cleared following cell death via the action of microglia, which is the hallmark of the neurodegenerative process in PD^2–4^. Through a combination of T1 and magnetization-transfer (MT) effects, NM can be estimated by MRI^5–7^.

The underlying basis for NM contrast in MRI is not entirely understood. NM contrast does not appear to arise solely from NM, but from a complex interplay of various properties of dopaminergic and noradrenergic neurons. These properties include a higher proton density and a lower macromolecular-to-free-water pool size ratio^6–8^. Despite the complexity of the underlying mechanism, NM-weighted imaging has consistently been correlated with histological dopaminergic cell loss in the substantia nigra pars compacta (SNpc)^2,4^. Additionally, several studies at 1.5/3T have shown that NM-sensitive imaging can help distinguish patients with PD from patients without PD^5,9–15^. NM imaging at standard field strengths, however, has a wide range of reported sensitivity (70–92%) and specificity (65–89%)^16^ Additionally, a meta-analysis found a sensitivity of only 82% and specificity of 82% in diagnosing PD at 3T^17^.

The limited performance of NM-sensitive imaging reported in the literature may partly be related to limitations of lower-field strength MRI, leading to poor signal-to-noise ratio (SNR) and NM contrast. To overcome these challenges, longer acquisition times and increased slice thickness are often required^18^, resulting in compromised spatial resolution for the small SNpc region, ultimately resulting in variable test-retest reliability^19–22^. While ultra-high-field MRI at 7T offers several advantages, including higher SNR and spatial resolution^23–28^, the higher specific absorption rate (SAR) at 7T, particularly for MT contrast, is a limiting factor that can diminish some of these advantages. There is currently limited evidence for the role of 7T NM imaging to diagnose PD from controls, and no studies comparing PD to essential tremor (ET). Recently, Wolters et al.^29^ assessed the NM signal intensity ratio in the SNpc and locus coeruleus using 7T MRI and found no significant differences between PD and healthy controls. These findings contradict prior 3T studies and histopathologic changes known to occur in PD, which may be related to limitations in the study design.

In our cross-sectional study, we demonstrate the use of NM imaging at 7T using a MT-prepared gradient-recalled echo (GRE) sequence^25^ due to its relatively lower SAR and consistent MT effect for each k-space line compared to alternatives^30^. Using the 7T MT-GRE sequence, we evaluate the performance of total SNpc volume, normalized contrast ratio (CR), and SNpc volume asymmetry index derived from NM-sensitive images as potential diagnostic biomarkers for PD. We also assess differences in NM between PD subtypes.

## Results

### Study demographics

There were 21 PD patients, 13 ET patients and 18 controls enrolled. Three control subjects were excluded: one due to a questionable history of dystonia, another for a previous cerebellar infarction, and the third for ataxia of uncertain origin. Additionally, one PD patient was excluded due to a ghosting artifact in the midbrain area. Patient demographics are shown in Table 1. There was no significant difference in age or gender distribution in all three cohorts. There was moderate correlation between total SNpc volume and disease duration, *r*(19) = −0.51, *p* = 0.02. No statistically significant correlation was found between total SNpc volume and H&Y stage, *r*(19) = −0.04, *p* = 0.86.

**Table 1:** Study demographics and clinical characteristics of study cohort.

Study participants	Parkinson’s disease	Essential tremor	Controls
Total (*n*)	21	13	18
Male (*n*, %)	17 (80.95%)	8 (61.54%)	13 (72.22%)
Female (*n*, %)	4 (19.05%)	5 (38.46%)	5 (27.78%)
Age at MRI (Mean ± SD) (years)	64.33 ± 10.86	61.08 ± 14.11	63.11 ± 13.34
Symptom duration (Mean ± SD) (years)	5.71 ± 3.51	18.5 ± 6.1	
Hoehn and Yahr scale			
1	9 (42.86%)		
2	7 (33.33%)		
3	5 (23.81%)		
Subtypes			
Akinetic-rigid	7 (33.33%)		
Tremor dominant	12 (57.14%)		
Postural instability/gait difficulty	2 (9.52%)		

### Parkinson’s disease versus patients without Parkinson’s disease

**Fig. 1:**
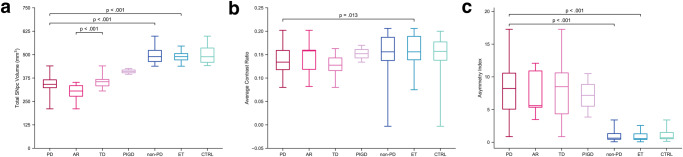
Distribution of measured variables across all groups. **a** Total SNpc volume in mm^3^, (**b**) average SNpc contrast ratio, and (**c**) asymmetry index are shown for patients with Parkinson’s disease (PD), including those with the akinetic-rigid subtype (AR), those with the tremor dominant subtype (TD), and those with postural instability/gait disturbance (PIGD), as well as all subjects without PD (non-PD), including those with essential tremor (ET) and control subjects (CTRL). Box plots illustrate the median and 25th and 75th percentiles, while whiskers represent minimum and maximum values. Statistically significant differences are indicated.

**Fig. 2:**
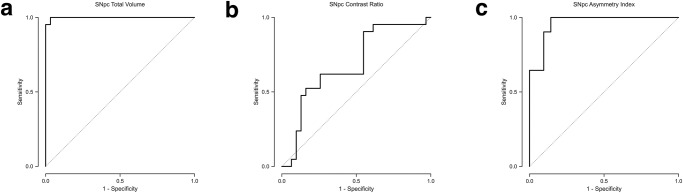
Differentiation of patients with Parkinson’s disease (PD) versus non-PD subjects. The receiver operating characteristic (ROC) analysis for identifying PD patients in a group consisting of both PD and non-PD subjects using (**a**) total substantia nigra pars compacta (SNpc) volume, (**b**) average SNpc contrast ratio (CR), and (**c**) asymmetry index. A total SNpc volume threshold of <440.6 mm^3^ yields an area under curve (AUC) of 0.998. An average CR threshold of <0.135 yields an AUC of 0.684. An asymmetry index threshold of >3.44 yields an AUC of 0.962.

Mean total SNpc volume in the PD cohort was 343.8 mm^3^ (SD = 53.0), lower than that in the non-PD cohort in which it was 493.2 mm^3^ (SD = 38.6). Our GLM analysis verified that the presence of PD as opposed to non-PD was a statistically significant predictor of decreased SNpc volume, *p* < 0.001 (Fig. 1a). Thresholding the SNpc total volume at <440.6 mm^3^ showed 100% sensitivity and 96.8% specificity (AUC = 0.998) for identifying PD in a group consisting of PD and non-PD subjects (Table 2 and Fig. 2a).

**Table 2:** Sensitivity and specificity of 7T neuromelanin measures in differentiating cohorts.

	PD vs. Non-PD		PD vs. ET		AR vs. TD	
	Sensitivity	Specificity	Sensitivity	Specificity	Sensitivity	Specificity
Total SNpc volume	100%	96.8%	95.2%	100%	100%	58.3%
Average contrast ratio	52.4%	83.9%	95.2%	46.2%	66.7%	71.4%
Asymmetry index	100%	85.7%	100%	85.7%	25.0%	100%

*PD* Parkinson’s disease, *ET* essential tremor, *Non-PD* controls and essential tremor cohort, *TD* tremor dominant PD, *AR* akinetic-rigid PD.

Average CR in the PD cohort was 0.13 (SD = 0.03), similar to that in the non-PD cohort in which it was 0.15 (SD = 0.04). The GLM analysis did not identify the presence of PD as opposed to non-PD as a statistically significant predictor of average CR, *p* = 0.07 (Fig. 1b). An average CR threshold of <0.135 had 52.4% sensitivity and 83.9% specificity (AUC = 0.684) for identifying PD in a group consisting of PD and non-PD subjects (Table 2 and Fig. 2b).

### Parkinson’s disease versus essential tremor

**Fig. 3:**
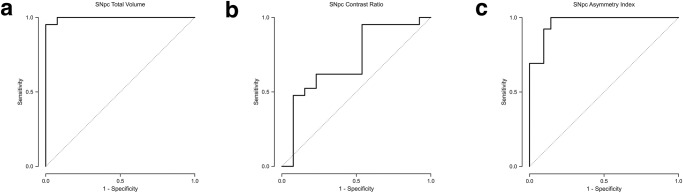
Differentiation of patients with Parkinson’s disease (PD) versus essential tremor (ET) subjects. The receiver operating characteristic (ROC) analysis for identifying PD patients in a group consisting of PD and ET subjects using (**a**) total substantia nigra pars compacta (SNpc) volume, (**b**) average SNpc contrast ratio (CR), and (**c**) asymmetry index. A total SNpc volume threshold of <432.1 mm^3^ yields an area under curve (AUC) of 0.996. An average CR threshold of <0.173 yields an AUC of 0.711. An asymmetry index threshold of >3.02 yields an AUC of 0.967.

Total SNpc volume in the PD cohort was 343.8 mm^3^ (SD = 53.0), lower than that in the ET cohort in which it was 488.5 mm^3^ (SD = 30.7). The presence of PD as opposed to ET was a statistically significant predictor of decreased SNpc volume, *p* < 0.001 (Fig. 1a). A total SNpc volume threshold of <432.1 mm^3^ had 95.2% sensitivity and 100% specificity (AUC = 0.996) for identifying PD in a group consisting of PD and ET subjects (Table 2 and Fig. 3a).

Average CR in the PD cohort was 0.13 (SD = 0.03), lower than that in the ET cohort in which it was 0.16 (SD = 0.04). The presence of PD as opposed to ET was a statistically significant predictor of decreased average CR, *p* = 0.013 (Fig. 1b). An average CR threshold of <0.173 had 95.2% sensitivity and 46.2% specificity (AUC = 0.711) for identifying PD in a group consisting of PD and ET subjects (Table 2 and Fig. 3b).

### Differentiating Parkinson’s disease subtypes

**Fig. 4:**
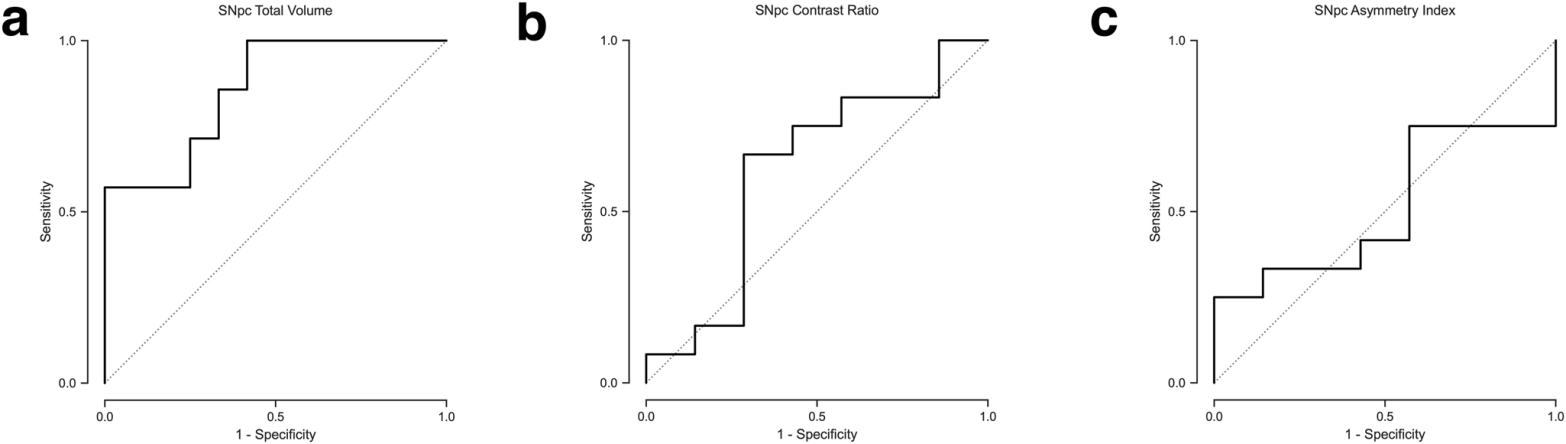
Differentiation of Parkinson’s disease (PD) subtypes. The receiver operating characteristic (ROC) analysis for identifying patients with akinetic-rigid (AR) Parkinson’s disease in a group consisting of AR and tremor dominant (TD) subjects using (**a**) total substantia nigra pars compacta (SNpc) volume, (**b**) average SNpc contrast ratio (CR), and (**c**) asymmetry index. A total SNpc volume threshold of <352.2 mm^3^ yields an area under curve (AUC) of 0.857. An average CR threshold of >0.134 yields an AUC of 0.619. An asymmetry index threshold of >2.83 yields an AUC of 0.512.

Total SNpc volume in PD patients with the AR subtype was 299.1 mm^3^ (SD = 49.1), lower than that in patients with the TD subtype in which it was 358.7 mm^3^ (SD = 37.8). The presence of the AR subtype as opposed to the TD subtype was a statistically significant predictor of decreased total SNpc volume, *p* < 0.001 (Fig. 1a). A total SNpc volume threshold of <352.2 mm^3^ had 100% sensitivity and 58.3% specificity (AUC = 0.857) for identifying PD patients with the AR subtype in a group consisting of AR and TD subtypes (Table 2 and Fig. 4a).

Average CR in PD patients with the AR subtype was 0.14 (SD = 0.04), similar to that in patients with the TD subtype in which it was 0.13 (SD = 0.03). The presence of the AR subtype as opposed to the TD subtype was not a statistically significant predictor of average CR, *p* = 0.58 (Fig. 1b). An average CR threshold of >0.134 had 66.7% sensitivity and 71.4% specificity (AUC = 0.619) for identifying patients with the AR subtype in a group consisting of AR and TD subtypes (Table 2 and Fig. 4b).

Due to a small number of subjects (*N* = 2), the PIGD subgroup was excluded from further analysis.

### Pattern of neuromelanin signal loss

**Fig. 5:**
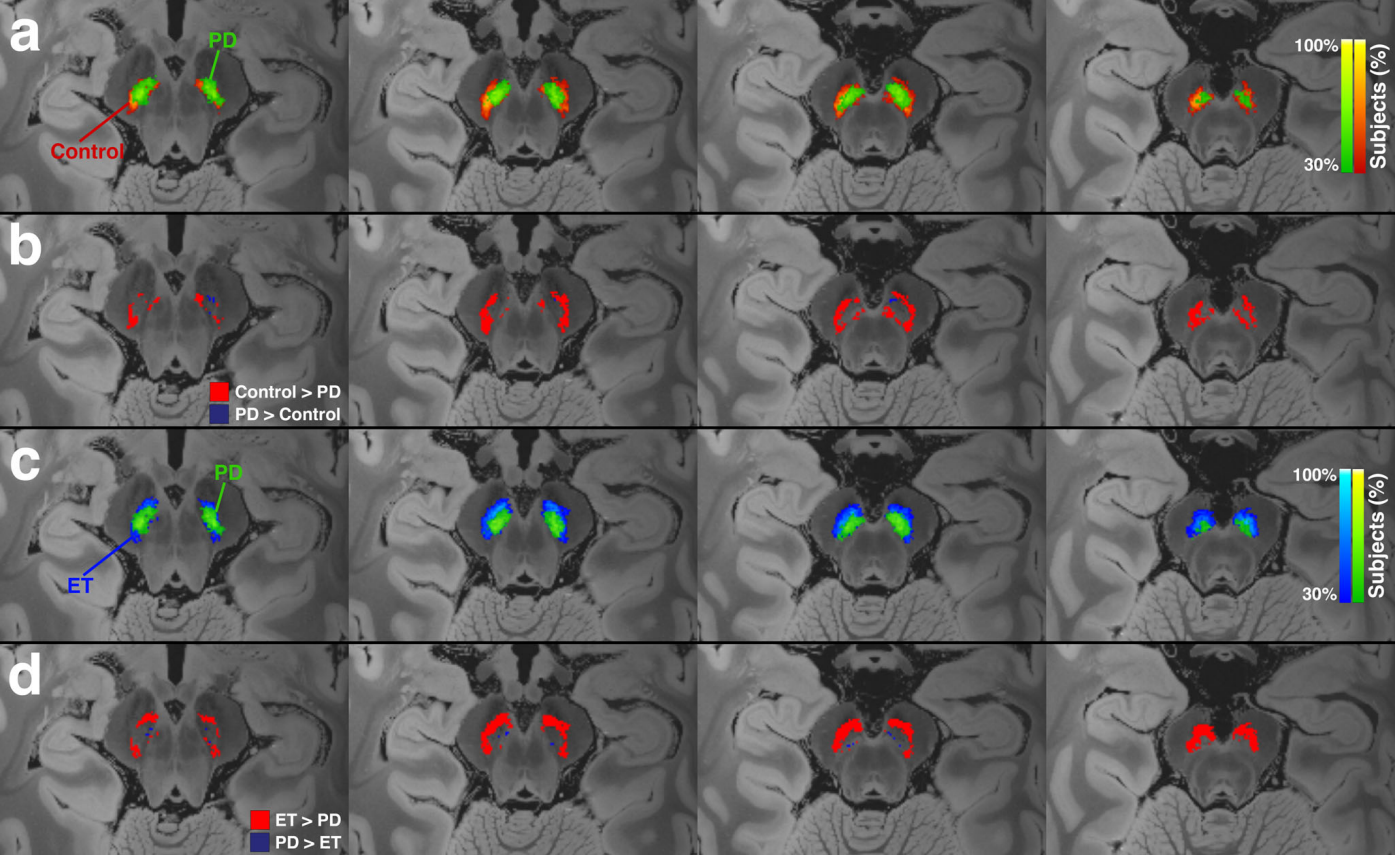
Normalized group mean substantia nigra pars compacta (SNpc) maps. **a** Heatmaps for the SNpc for the PD (green) and control (red–orange). **b** SNpc subtraction maps showing voxels with greater SNpc in controls (red) and greater SNpc in PD (purple). **c** Heatmaps for the SNpc for the PD (green) and ET (blue). **d** SNpc subtraction maps showing voxels with greater SNpc in ET (red) and greater SNpc in PD (purple). Neuromelanin (NM) pigment loss in PD was more pronounced along the lateral and inferior aspect of SNpc compared to both ET and control groups.

**Fig. 6:**
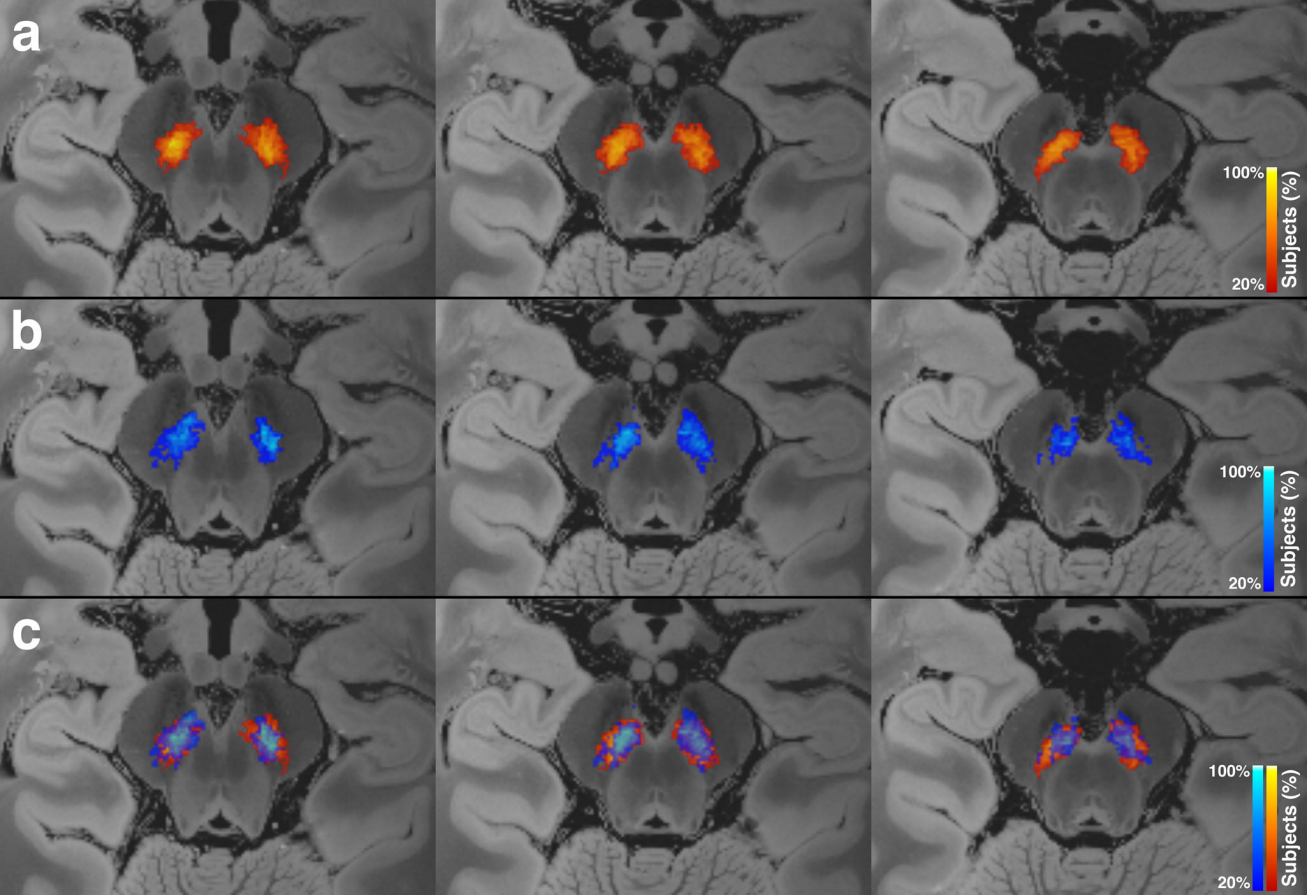
Normalized group mean substantia nigra pars compacta (SNpc) maps. Heatmaps for the SNpc for the (**a**) tremor dominant (TD) and (**b**) akinetic-rigid (AR) subgroups. **c** Overlay of SNpc maps for TD (orange) and AR (blue) showing greater NM pigment loss in the AR subgroup, most pronounced along the lateral and inferior aspect of SNpc.

There was a distinct imaging pattern of NM signal loss in the SNpc of patients with PD as compared to ET and control. The NM signal loss was more pronounced along the lateral, posterior, and inferior aspects of SNpc in PD (Fig. 5). A similar pattern was observed between TD and AR, with NM signal loss primarily affecting the lateral, posterior, and inferior aspects of SNpc in the AR subgroup as opposed to TD (Fig. 6).

Additionally, there was asymmetric involvement of the SNpc in PD patients. The average SNpc volume asymmetry index in PD patients was 7.75 (SD = 4.47), greater than that in the ET group in which it was 0.79 (SD = 0.68), and also greater than that of the aggregate non-PD group in which it was 0.96 (SD = 0.84). The presence of PD as opposed to either ET or non-PD was a statistically significant predictor of a higher asymmetry index, *p* < 0.001 in both cases (Fig. 1c). An asymmetry index threshold of >3.44 had 100% sensitivity and 85.7% specificity (AUC = 0.962) for identifying PD in a group consisting of PD and non-PD subjects (Table 2 and Fig. 2c). An asymmetry threshold of >3.02 had 100% sensitivity and 85.7% specificity (AUC = 0.967) for identifying PD in a group consisting of PD and ET subjects (Table 2 and Fig. 3c).

Considering the PD subtypes, the average SNpc volume asymmetry index in the AR group was 7.67 (SD = 3.49), similar to that in the TD group in which it was 7.89 (SD = 5.25). The presence of the AR subtype as opposed to the TD subtype was not a statistically significant predictor of the asymmetry index, *p* = 0.67. An asymmetry index threshold of >2.83 had 25.0% sensitivity and 100% specificity for identifying patients with the AR subtype in a group consisting of AR and TD subtypes (AUC = 0.512) (Table 2 and Fig. 4c).

## Discussion

We have shown that 7T NM imaging is a valuable tool in the diagnosis of PD. To our knowledge, this is the first study to assess 7T NM imaging in PD versus both controls and ET. We found that 7T has greater sensitivity and specificity for differentiating PD from non-PD patients than commonly reported for 3T NM imaging, which is likely due to higher SNR and spatial resolution of 7T. Similar to prior studies at 3T, we found that NM SNpc volume performs better than CR in the diagnosis of PD, which provides an easily adaptable tool to clinical practice. Lastly, we also show significantly greater NM signal loss in AR versus TD subtypes. NM imaging at 7T is a promising tool for future studies that may allow prediction of symptom-specific disease models in PD.

Prior 3T studies have shown a wide range of sensitivity and specificity, ranging from 70–90.6% and 65–89% with AUC typically <0.9^31–35^. Additionally, a meta-analysis reported a sensitivity of 82% and specificity of 82% in diagnosing PD at 3T^17^. Few 3T studies have compared PD and ET cohorts with NM imaging, but many with relatively low AUC^36–39^. The best outcomes reported were by Jin et al.^40^ with an AUC of 0.89, sensitivity of 88%, and specificity of 80% in differentiating PD from ET. Nevertheless, there is continued interest in NM imaging due to its potential superiority over other MRI biomarkers, such as the dorsal nigral hyperintensity, or so-called “swallow tail sign”^40,41^.

One advantage to 7T NM imaging is the higher SNR, allowing improved spatial resolution. Our protocol used a 0.24 mm^3^ voxel volume, an ~40–73% decrease in voxel volume compared to 3T which ranges from 0.4–0.9 mm^3^. Studies using variable voxel sizes at 3T have reported a wide range of normal and PD volumes with mean total volumes in normal ranging from ~257–706 mm^3^ and PD from 83–474 mm^3 ^^32–35,42^. This suggests that partial volume averaging or other sequence factors play a major effect on volume measurements. 7T also has advantageous tissue relaxation properties at 7T for NM imaging. The shortened tissue T2 relaxation time, coupled with the broader spectrum of macromolecules, provides a wide array of off-resonance frequencies that can be utilized for MT pulses. Furthermore, extended T1 relaxation time and prolonged longitudinal Mz recovery afford a more effective saturation transfer between protons in the macromolecular pool and the free water pool. These advantages on MT effects could still be impaired by the substantially increased SAR at 7T. Therefore, careful optimization of pulse sequence parameters, the MT pulse, and acquisition strategy are important for 7T NM imaging. Several MRI acquisition strategies have been proposed for NM contrast. In this study, we opted for a non-segmented, MT-prepared GRE sequence that is readily available as a product sequence, with relatively low SAR versus other commonly used NM-imaging approaches. Conversely, a turbo spin echo sequence augmented by MT pulses often resulted in excessively high SAR, rendering it impractical for NM imaging^43^. The turbo-FLASH sequence, a segmentation-based acquisition featuring MT pulses before an echo train, has also been utilized for NM imaging^29^. Furthermore, we minimized the TE to mitigate T2* weighting and preserve MT contrast. Owing to the implementation of MT pulses, a longer TR was necessitated to comply with the more challenging SAR constraint at 7T, which facilitated the inclusion of a multi-echo capability to simultaneously generate NM, R2*, and susceptibility-weighted images that can leverage the combined diagnostic ability of NM, iron concentration, and the dorsal nigral hyperintensity sign^25,41^.

To our knowledge, there is only one prior study examining the diagnostic performance of 7T NM imaging for differentiating PD from controls, which found no difference in SNpc CR^29^. We found no significant CR difference in PD versus non-PD cohorts. This is not surprising since prior 3T studies have also repeatedly shown that CR underperforms compared to SNpc volume in discriminating PD from controls^33,35^. Isaias et al. found that CR contradicted dopamine transporter density in 39% of cases, and the best predictor of dopamine transporter density was the SNpc volume^33^. There are several potential reasons for the limitations in CR, including the SNpc contrast itself. For example, in the prior study, the authors utilized a longer echo time (4.08 ms vs. 2.18 ms), as well as a turbo-FLASH sequence featuring MT pulses only before an echo train, both of which could lead to a progressive loss of NM weighting^29^. However, this can be alleviated by carefully optimizing the MT pulse train and the turbo-FLASH segmentation. The benefit of our approach to sequence optimization can be seen by the higher SNpc contrast in our images compared to Wolters et al.^29^, as evidenced by the higher average CR in our cohorts.

We found that asymmetric loss of NM signal of SNpc is a specific biomarker for differentiating PD from patients without PD. This observation is in line with histopathological studies showing asymmetric degeneration of NM pigment of the SNpc^44^. The lower AUC versus total SNpc volume is not surprising since not all PD patients have asymmetric disease^45^. The α-Synuclein Origin and Connectome Model of PD posits that symmetry of PD pathology in the SN is predicted by the origin of the α-synuclein pathology, with “brain-first” PD originating and spreading initially within one hemisphere, as opposed to the “body-first” group originating in the gut and spreading to both SN via the crossing dorsal motor nuclei of the vagus nerve^45^. In contrast, atypical parkinsonian syndromes more frequently present with symmetric NM loss, and NM imaging may be a valuable tool in distinguishing from PD.

We also identified a distinct imaging pattern of NM signal loss in PD, which was more pronounced along the inferior, posterior, and lateral aspects of SNpc. This distinct pattern of imaging is concordant with the histological studies showing cell loss greater in the nigrosomes with an overall lateral-to-medial, caudal-to-rostral, and ventral-to-dorsal progression^46^. This is also supported by MRI data showing that nigrosome 1 is the most affected subregion in patients with PD (located in the lateral-ventral portion of SNpc)^47^. Few other studies of NM-sensitive imaging at lower field strength have shown reduction in NM signal along the lateral-ventral regions of SNpc^18,31,34^, which aligns with our observation.

Furthermore, we found that the degree of NM signal loss in SNpc was greater in the AR than TD subtype and primarily affected the ventrolateral aspect in the AR subgroup. Our observation agrees with autopsy results showing greater loss of NM-pigmented cells in SNpc in patients with non-TD subtypes compared to TD^48,49^. In particular, there is greater degeneration of ventrolateral SNpc which connects to the dorsal putamen resulting in striato-thalamo-cortical dysfunction^50^ and inhibition of the direct pathway^51^. These findings corroborate pathophysiological mechanisms with tremor being mediated by a cerebello-thalamo-cortical network rather than nigrostriatal pathways^52^. Histologically, SNpc cell loss also correlates with severity of AR symptoms^53^. In combination, our findings may help explain mixed results in studies assessing correlation of clinical severity and SNpc volume on NM imaging. When subtypes of PD are mixed, TD patients with more severe symptoms may not show as great of SNpc volume reduction as non-TD patients with similar severity. Despite clear histopathological evidence, there are varying reports of NM imaging performance in the literature, such as a study by Wang et al.^37^ in de novo Parkinson disease showing no significant difference between PD subgroups.

There are some limitations to our study, such as the relatively small sample size and retrospective nature. We included subjects with an established clinical diagnosis of PD. Further studies are needed to assess patients in premotor stages, equivocal PD, or atypical parkinsonism. Additionally, the MT-GRE sequence utilized herein does have certain limitations. The MT pulse was a fixed-amplitude MTC pulse with a specific offset frequency, thereby constraining the ability to fine-tune MT pulse parameters for optimal MT effects. Furthermore, the choice of MT pulse, and the strategy of implementing MT pulses are often restricted by SAR limit at 7T. Due to the extended TR (55 ms), the initial echo exhibited more proton density weighting, while the hyperintense T1 shortening effects were diminished despite the paramagnetic NM-bound iron. Consequently, the PD weighting led to CSF hyperintensity, which could potentially hinder evaluation of the adjacent locus coeruleus. Lastly, accurate normalization of brainstem nuclei, particularly in the pathologic state, has proven challenging^54^. As such, results from group-level analyses in MNI space may be subject to normalization inaccuracies, which further supports our approach of patient-specific volumetric measurements.

7T NM imaging is a promising biomarker in the diagnosis of PD, but currently with limited clinical adoption. Higher SNR and spatial resolution at 7T may be advantageous in increasing diagnostic performance. Future studies are needed to further optimize NM imaging sequences at 7T, as well as show its performance across a wide range of parkinsonian syndromes and mimics.

## Methods

### Study design and cohort

This retrospective study was granted an exemption for requirement of documented consent by the Mayo Clinic Institutional Review Board. A series of consecutive patients undergoing 7T MRI (from 2021–2023) with a diagnosis of levodopa-responsive PD by the Mayo Clinic Florida Movement Disorders Clinic was retrospectively analyzed. Patients meeting the Movement Disorder Society guidelines^55^ for the diagnosis of Clinically Established PD were included. All charts were reviewed at the time of the study for any new clinical symptoms or additional information that may suggest an alternative non-PD diagnosis or development of dementia within 1 year from onset (e.g., dementia with Lewy bodies).

Patients were further subclassified as tremor-dominant (TD) if tremor was the most prominent clinical feature, akinetic-rigid (AR) if rigidity and bradykinesia were the prominent features, or postural instability/gait difficulty (PIGD) if gait difficulty was the most prominent feature^56^. Basic clinical and demographic information was collected, including age, sex, disease duration, and Hoehn and Yahr (H&Y) stage.

A cohort of ET patients diagnosed according to the guidelines of the International Parkinson and Movement Disorder Society^57^ were also selected from a consecutive series of patients undergoing preoperative 7T MRI for deep brain stimulator placement. Lastly, a control group was selected from a consecutive series of patients who received 7T NM-MRI as part of a routine imaging protocol that was matched for age and sex. Control patients were excluded for any signs or symptoms of a movement disorder, gross structural brain abnormalities, or other neurodegenerative disorders. Subjects with severe motion artifacts, ghosting artifacts, or other image quality issue were excluded from further analysis. A non-PD cohort was also established consisting of the combined control and ET cohorts.

### Image acquisition

Patients were scanned on a clinical 7T MRI scanner (Magnetom Terra, Siemens Healthineers, Erlangen, Germany) equipped with an 8-channel transmit 32-channel receive head coil (Nova Medical Inc, Wilmington, MA) operating in circularly polarized transmission mode (“TrueForm”). The MRI scan included a NM-sensitive multi-echo 3D GRE sequence, as well as an optimized T1-weighted MP2RAGE sequence for anatomical reference (Table 3)^58^. The NM-sensitive sequence was an axial multi-echo 3D GRE sequence (matrix size = 256 × 192, FOV = 200 × 150, number of slices = 88, no partial Fourier) including a product MT pulse (Gaussian pulse: FA = 500°, frequency offset = 1200 Hz, BW = 192 Hz, duration = 9.98 ms) with other parameters listed in Table 3.

**Table 3:** MRI sequence parameters for neuromelanin-weighted imaging and MP2RAGE.

Sequence	TR (ms)	TE (ms)	FA (deg)	TI (ms)	Resolution (mm)	iPAT	BW (Hz/Px)	TA (m:s)
NM-weighted	55	2.18/4.15/7.09	16°	–	0.4 × 0.4 × 1.5 (0.8 × 0.8 × 1.5 without interpolation)	3	570	7:16
MP2RAGE	4500	2.21	5°/4°	800/2700	0.8 × 0.8 × 0.8	3	200	9:02

*NM* neuromelanin, *MP2RAGE* magnetization prepared rapid acquisition gradient echo with two inversions, *TR* repetition time; *TE* echo time, *TI* inversion time, *FA* flip angle, *iPAT* integrated parallel acquisition techniques, *BW* bandwidth, *TA* time of acquisition.

### Image analysis

The first echo image (TE = 2.2 ms) was used for its NM weighting. The NM-MRI was manually segmented as the hyperintense signal within the SNpc area, excluding the midline VTA nucleus. Segmentation was performed by a board-certified neuroradiologist with 8 years of experience in diagnosing and treating movement disorders, who was blinded to the subject diagnosis. Segmentation was performed in ITK-SNAP (http://www.itksnap.org/). This method has been shown to be highly reproducible^41^. Left, right, and total SNpc volumes were calculated for all subjects.

Next, the NM-MRI was co-registered to the anatomic T1-weighted MP2RAGE UNI image using Statistical Parametric Mapping (SPM) v12 (https://www.fil.ion.ucl.ac.uk/spm/software/spm12/). The T1-weighted volume was then normalized to MNI_ICBM_2009b_NLIN_ASYM template space using Advanced Normalization Tools (ANTs) (http://stnava.github.io/ANTs/) and the resulting warp was applied to the coregistered NM-MRI. A region-of-interest(ROI) for the SNpc signal was adapted from the HybraPD Atlas^59^. An ROI for the left and right cerebral peduncle (CP) was manually drawn on the MNI template encompassing the CP at the level of the midpoint of the SN. Similar to prior studies, a normalized SNpc CR was calculated for each side using equation (1): CR = (SNpc − CP)/CP, where SNpc = mean signal intensity of the SNpc ROI and CP = mean signal of the ipsilateral cerebral peduncle ROI. An average CR was calculated for each subject using the right and left sides. An asymmetry index was calculated for the SNpc volume using equation (2): Asymmetry Index = |(SNpc_LEFT_ − SNpc_RIGHT_)/(SNpc_LEFT_ + SNpc_RIGHT_)| × 100.

Lastly, the normalized SNpc masks were averaged across each subgroup to illustrate patterns of NM loss within the SNpc. The masks were also binarized for all voxels with SNpc in >30% of subjects and used to create subtraction masks between the PD and control groups and the PD and ET groups.

To assess for differences in patient motion, an average edge strength (AES) was calculated for each subjects’ NM-weighted exam using a previously described method^60^. AES assesses image blurring as a surrogate of motion in structural imaging by quantifying the average contrast intensity at edges within the image. AES has been shown to be sensitive to head motion in MRI and in assessing differences in motion between PD patients and controls^60^.

### Statistical analysis

Data analysis was performed using the Python statsmodels package, version 0.14.0. Descriptive statistics were used to summarize data including mean, standard deviation, and number (percentage). Outcome variables were depicted graphically with box and whisker plots. Differences in the image-derived metrics were assessed using a general linear model (GLM) analysis with significance level of *p* < .05. Group comparisons were limited to those scenarios that are clinically meaningful: identifying PD in the general population, distinguishing PD from ET, and differentiating between PD subtypes. The model included age, sex, and AES as regressors to control for the effects of these variables.

To evaluate diagnostic performance, we plotted receiver operating characteristic (ROC) curves and computed the area under the curve (AUC) for each ROC plot. The optimized sensitivity and specificity for each ROC curve were determined using Youden’s index.

### Reporting summary

Further information on research design is available in the Nature Research Reporting Summary linked to this article.

### Supplementary information

Reporting Summary

STROBE Checklist

## Data Availability

The data are not publicly available due to the inclusion of information that could compromise the participants’ privacy. The datasets used during the study are available from the corresponding author on request with institutional review board approval.
