# Supplementary material for: Diagnostic utility of 7T neuromelanin imaging of the substantia nigra in Parkinson’s disease
Source: NPJ Parkinsons Dis. 2024 Jan 8;10:13. doi: 10.1038/s41531-024-00631-3 (PMC10774294; doi:10.1038/s41531-024-00631-3)
Supplement: Supplementary file 1 — Reporting Summary [file 41531_2024_631_MOESM1_ESM.pdf]

nature portfolio

Corresponding author(s): Erik H. Middlebrooks, MD

Last updated by author(s): Nov 29, 2023

Reporting Summary

Nature Portfolio wishes to improve the reproducibility of the work that we publish. This form provides structure for consistency and transparency in reporting. For further information on Nature Portfolio policies, see our Editorial Policies and the Editorial Policy Checklist.

Statistics

For all statistical analyses, confirm that the following items are present in the figure legend, table legend, main text, or Methods section.

n/a	Confirmed	
☐	☒	The exact sample size (*n*) for each experimental group/condition, given as a discrete number and unit of measurement
☒	☐	A statement on whether measurements were taken from distinct samples or whether the same sample was measured repeatedly
☐	☒	The statistical test(s) used AND whether they are one- or two-sided<br>*Only common tests should be described solely by name; describe more complex techniques in the Methods section.*
☐	☒	A description of all covariates tested
☐	☒	A description of any assumptions or corrections, such as tests of normality and adjustment for multiple comparisons
☐	☒	A full description of the statistical parameters including central tendency (e.g. means) or other basic estimates (e.g. regression coefficient) AND variation (e.g. standard deviation) or associated estimates of uncertainty (e.g. confidence intervals)
☐	☒	For null hypothesis testing, the test statistic (e.g. *F*, *t*, *r*) with confidence intervals, effect sizes, degrees of freedom and *P* value noted<br>*Give P values as exact values whenever suitable.*
☒	☐	For Bayesian analysis, information on the choice of priors and Markov chain Monte Carlo settings
☒	☐	For hierarchical and complex designs, identification of the appropriate level for tests and full reporting of outcomes
☒	☐	Estimates of effect sizes (e.g. Cohen's *d*, Pearson's *r*), indicating how they were calculated

Our web collection on statistics for biologists contains articles on many of the points above.

Software and code

Policy information about availability of computer code

Data collection	No custom software was used.
Data analysis	No custom software was used.

For manuscripts utilizing custom algorithms or software that are central to the research but not yet described in published literature, software must be made available to editors and reviewers. We strongly encourage code deposition in a community repository (e.g. GitHub). See the Nature Portfolio guidelines for submitting code & software for further information.

Data

Policy information about availability of data

All manuscripts must include a data availability statement. This statement should provide the following information, where applicable:

- Accession codes, unique identifiers, or web links for publicly available datasets
- A description of any restrictions on data availability
- For clinical datasets or third party data, please ensure that the statement adheres to our policy

The datasets used and/or analyzed during the current study available from the corresponding author on reasonable request.

Research involving human participants, their data, or biological material

Policy information about studies with human participants or human data. See also policy information about sex, gender (identity/presentation), and sexual orientation and race, ethnicity and racism.

Reporting on sex and gender	Sex and gender were not specific attributes in the study design (inclusion/exclusion). Consecutive patients were chosen from the cohorts and representative of the distribution of disease in our population. Sex was included as a regressor in statistical models to account for differences related to study findings.
Reporting on race, ethnicity, or other socially relevant groupings	Race or ethnicity were not specific attributes in the study design (inclusion/exclusion). Consecutive patients were chosen from the cohorts and representative of the distribution of disease in our population. This information was not collected on study subjects.
Population characteristics	Study population was derived from the disease-specific cohorts and representative of the distribution of disease in our population. No specific recruitment of demographics was applied.
Recruitment	Consecutive patients were chosen from the cohorts and representative of the distribution of disease in our population.
Ethics oversight	Mayo Clinic

Note that full information on the approval of the study protocol must also be provided in the manuscript.

Field-specific reporting

Please select the one below that is the best fit for your research. If you are not sure, read the appropriate sections before making your selection.

☒ Life sciences ☐ Behavioural & social sciences ☐ Ecological, evolutionary & environmental sciences

For a reference copy of the document with all sections, see nature.com/documents/nr-reporting-summary-flat.pdf

Life sciences study design

All studies must disclose on these points even when the disclosure is negative.

Sample size	This first-in-human test of a novel MRI sequence did have a power analysis due to lack of existing pilot data.
Data exclusions	*Describe any data exclusions. If no data were excluded from the analyses, state so OR if data were excluded, describe the exclusions and the rationale behind them, indicating whether exclusion criteria were pre-established.*
Replication	Patient exclusions were based on pre-determined criteria of clinical disease states, imaging artifacts, or unrelated brain structural changes that could impact imaging results. The exclusion criteria are explicitly stated, as well as details for each excluded subject.
Randomization	Not applicable--retrospective cross-sectional study.
Blinding	Expert reader blinded to clinical diagnosis.

Reporting for specific materials, systems and methods

We require information from authors about some types of materials, experimental systems and methods used in many studies. Here, indicate whether each material, system or method listed is relevant to your study. If you are not sure if a list item applies to your research, read the appropriate section before selecting a response.

Materials & experimental systems

n/a	Involved in the study	
☒	☐	Antibodies
☒	☐	Eukaryotic cell lines
☒	☐	Palaeontology and archaeology
☒	☐	Animals and other organisms
☒	☐	Clinical data
☒	☐	Dual use research of concern
☒	☐	Plants

Methods

n/a	Involved in the study	
☒	☐	ChIP-seq
☒	☐	Flow cytometry
☐	☒	MRI-based neuroimaging

Plants

Seed stocks	*Report on the source of all seed stocks or other plant material used. If applicable, state the seed stock centre and catalogue number. If plant specimens were collected from the field, describe the collection location, date and sampling procedures.*
Novel plant genotypes	*Describe the methods by which all novel plant genotypes were produced. This includes those generated by transgenic approaches, gene editing, chemical/radiation-based mutagenesis and hybridization. For transgenic lines, describe the transformation method, the number of independent lines analyzed and the generation upon which experiments were performed. For gene-edited lines, describe the editor used, the endogenous sequence targeted for editing, the targeting guide RNA sequence (if applicable) and how the editor was applied.*
Authentication	*Describe any authentication procedures for each seed stock used or novel genotype generated. Describe any experiments used to assess the effect of a mutation and, where applicable, how potential secondary effects (e.g. second site T-DNA insertions, mosiacism, off-target gene editing) were examined.*

Magnetic resonance imaging

Experimental design

Design type	3D MT GRE Neuromelanin-weighted imaging
Design specifications	N/A
Behavioral performance measures	N/A

Acquisition

Imaging type(s)	Structural
Field strength	7
Sequence & imaging parameters	The NM-sensitive sequence was an axial multi-echo 3D GRE sequence (matrix size=256 x 192, FOV=200 x 150, number of slices = 88, no partial Fourier) including a product MT pulse (Gaussian pulse: FA=500°, frequency offset=1200Hz, BW=192Hz, duration=9.98ms) with other parameters listed in Table 1.
Area of acquisition	Whole brain

Diffusion MRI ☐ Used ☒ Not used

Preprocessing

Preprocessing software	Segmentation was performed in ITK-SNAP (http://www.itksnap.org/).
Normalization	NM-MRI was co-registered to the anatomic T1-weighted MP2RAGE UNI image using Statistical Parametric Mapping (SPM) v12 (https://www.fil.ion.ucl.ac.uk/spm/software/spm12/). The T1-weighted volume was then normalized to MNI_ICBM_2009b_NLIN_ASYM template space using Advanced Normalization Tools (ANTs)(http://stnava.github.io/ANTs/) and the resulting warp was applied to the coregistered NM-MRI. Default settings were used.
Normalization template	MNI_ICBM_2009b_NLIN_ASYM template
Noise and artifact removal	N/A
Volume censoring	N/A

Statistical modeling & inference

Model type and settings	N/A
Effect(s) tested	N/A

Specify type of analysis: ☐ Whole brain ☐ ROI-based ☐ Both

Statistic type for inference (See Eklund et al. 2016)	N/A
Correction	N/A

Models & analysis

n/a	Involved in the study
☒	☐ Functional and/or effective connectivity
☒	☐ Graph analysis
☒	☐ Multivariate modeling or predictive analysis
